# Determinants of Quality of Life Among Community-Dwelling Older Adults in Urban South India: A Community-Based Cross-Sectional Study

**DOI:** 10.7759/cureus.114814

**Published:** 2026-08-19

**Authors:** Parthiban Ravichandiran, Sooraj Vinayakumar, Ajo Paul, Gamani Vellore Indrasen

**Affiliations:** 1 Department of Community Medicine, Arunai Medical College and Hospital, Tiruvannamalai, IND; 2 Department of Community Medicine, Sree Narayana Institute of Medical Sciences, Ernakulam, IND; 3 Department of Community Medicine, GITAM Institute of Medical Sciences and Research, Visakhapatnam, IND

**Keywords:** community-dwelling older adults, elderly population, geriatrics population, healthy ageing, quality of life (qol), social determinants of health (sdoh), urban india, whoqol-bref

## Abstract

Background

Population ageing poses significant public health challenges, particularly in low- and middle-income countries. India’s ageing population is rapidly increasing, posing challenges to quality of life (QoL) because of declining physical, functional, and psychological well-being. This study aimed to assess QoL and identify its determinants among the older urban population in Visakhapatnam, India.

Methods

A community-based cross-sectional study was conducted among 400 older adults (≥60 years) selected by simple random sampling from the electoral roll of an urban field practice area in Visakhapatnam, India. Data were collected through face-to-face interviews using a pretested questionnaire and the Telugu version of the World Health Organization Quality of Life-BREF (WHOQOL-BREF). QoL scores were calculated according to WHO guidelines, and determinants of QoL were analyzed using descriptive statistics, an independent-samples *t*-test, one-way ANOVA, and multiple linear regression.

Results

The mean overall QoL score was 37.03 (SD = 10.35). The domain-specific mean scores were 39.44 (SD = 13.12) for physical health, 41.34 (SD = 13.14) for psychological health, 27.08 (SD = 8.88) for social relationships, and 40.24 (SD = 11.42) for the environment. Of the 400 participants, 62 (15.5%) had poor QoL, 236 (59.0%) had average QoL, and 102 (25.5%) had good QoL. In bivariate analyses, higher QoL was significantly associated with female sex, higher education, upper-middle socioeconomic status, involvement in household decision-making, and enrolment in welfare schemes for older adults. Increasing age was independently associated with lower overall QoL (β = -0.13, *p* = 0.013). Multiple linear regression identified increasing age, education, socioeconomic status, enrolment in welfare schemes for older adults, and participation in household decision-making as independent determinants of overall QoL.

Conclusion

Most older adults had an average QoL, and the social relationships domain had the lowest mean score. Educational attainment, socioeconomic status, participation in social welfare schemes, and decision-making autonomy were important determinants of better QoL. Interventions that strengthen social support, improve access to welfare programmes, and address socioeconomic inequalities may contribute to healthy ageing and improved QoL among older adults.

## Introduction

Population ageing is a defining demographic trend of the twenty-first century. Improvements in healthcare, nutrition, sanitation, and socioeconomic conditions have led to substantial increases in life expectancy, resulting in a rapidly expanding older population worldwide [1]. The United Nations projects that the number and proportion of older persons will continue to rise over the coming decades, posing significant challenges to health systems and social care. As populations age, the burden of chronic noncommunicable diseases, multimorbidity, disability, and functional dependence also increases, shifting the focus of healthcare from extending survival to promoting healthy ageing and preserving functional ability [1,2].

India is experiencing a similar demographic transition. According to the India Ageing Report 2023, the population aged 60 years and above increased from 104 million in 2011 to approximately 149 million in 2022 and is projected to reach 347 million by 2050, accounting for approximately one-fifth of the country’s population [3]. Findings from the Longitudinal Ageing Study in India (LASI), the first nationally representative longitudinal survey of middle-aged and older adults, further demonstrate the high prevalence of chronic diseases, functional limitations, and social and economic vulnerabilities among older adults in India [4]. These demographic and epidemiological changes highlight the growing need for evidence-based strategies to improve health and well-being in later life.

Quality of life (QoL) is increasingly recognised as a key outcome of healthy ageing. Beyond the absence of disease, it reflects an individual’s perception of physical health, psychological well-being, social relationships, and environmental circumstances. The WHO defines QoL as an individual’s perception of their position in life within the context of the culture and value systems in which they live and in relation to their goals, expectations, standards, and concerns [5]. To facilitate cross-cultural assessment, the WHO developed the World Health Organization Quality of Life-BREF (WHOQOL-BREF), a validated instrument that has been widely used across diverse populations and settings [6].

Several factors, including age, educational attainment, socioeconomic status, chronic illnesses, social support, and participation in decision-making, have been shown to influence QoL among older adults. Understanding these determinants is important for identifying vulnerable groups, informing community-based interventions, strengthening primary healthcare services, and guiding policies aimed at promoting healthy ageing. In this context, assessing QoL among community-dwelling older adults provides valuable evidence for planning comprehensive geriatric health programmes.

The present study aimed to assess QoL and identify its determinants among community-dwelling older adults aged 60 years and above residing in the urban field practice area of a tertiary care teaching hospital in Visakhapatnam, Andhra Pradesh, India.

## Materials and methods

Study design and setting

A community-based cross-sectional study was conducted from March 2023 to March 2025 among older adults residing in the urban field practice area attached to a tertiary care teaching hospital in Visakhapatnam, Andhra Pradesh, India.

Study participants

Individuals aged 60 years and above who had resided in the study area for at least six months were eligible for inclusion. The detailed inclusion and exclusion criteria are presented in Table 1.

**Table 1:** Inclusion and exclusion criteria for study participants.

Inclusion criteria	Exclusion criteria
Age ≥60 years	Critical illness preventing participation
Residence in the study area for ≥6 months	Severe hearing, visual, or speech impairment affecting communication
Willingness to provide informed consent	Diagnosed psychiatric illness (e.g., schizophrenia) or intellectual disability
	Neurological disorder affecting communication (e.g., parkinsonism)
	Acute illness at the time of the interview

Sample size estimation

The sample size was estimated using the formula for estimating a single population mean: (n = \frac{Z_{1-\alpha/2}^{2}\sigma^{2}}{d^{2}}), where (n) is the required sample size, (Z) is the standard normal deviate corresponding to a 95% confidence level (1.96), (\sigma) is the SD of the outcome variable, and (d) is the absolute precision.

The SD ((\sigma = 9.7)) was obtained from the social relationships domain of the WHOQOL-BREF reported in a community-based study conducted among older adults in an urban area of Ahmedabad, India [7]. An absolute precision of 1.0 QoL unit was considered appropriate. Based on these assumptions, the minimum required sample size was calculated as 362 participants. After incorporating a 10% allowance for nonresponse, the final sample size was rounded to 400 participants.

Sampling procedure

Simple random sampling was employed. The updated 2022 electoral roll for the study area, obtained from the local administrative office (Sachivalayam), served as the sampling frame. All residents aged 60 years and above were identified and enumerated, and each eligible individual was assigned a unique identification number. A computerized random number generator was used to select 400 participants.

Selected participants who were unavailable during the initial visit were revisited on three separate occasions at different times before being considered nonresponders. When a selected participant remained unavailable after three visits, the next eligible individual in the sampling frame was recruited to maintain the predetermined sample size.

Study instrument

Data were collected using a pretested, semi-structured, interviewer-administered questionnaire consisting of two components. The first component collected information on sociodemographic and socioeconomic characteristics, including age, sex, education, marital status, family type, occupation, source of income, socioeconomic status, enrolment in government welfare schemes for older adults, and participation in household decision-making. The second component comprised the WHOQOL-BREF questionnaire for assessing QoL.

WHOQOL-BREF questionnaire

Permission to use and translate the WHOQOL-BREF questionnaire was obtained from the WHO before the commencement of the study (WHO Permission Request No. 392355). The Telugu version used in this study was translated and culturally adapted according to the WHO translation methodology, including forward translation, expert review, and pretesting to ensure conceptual equivalence and cultural appropriateness.

The WHOQOL-BREF is a validated instrument containing 26 items, including two general questions on overall QoL and overall health and 24 items distributed across four domains: physical health, psychological health, social relationships, and environment. Each item is scored on a five-point Likert scale. Raw domain scores were calculated according to the WHOQOL-BREF scoring manual and subsequently transformed to a 0-100 scale, with higher scores indicating better QoL [5].

The internal consistency of the translated instrument was assessed, yielding a Cronbach’s alpha coefficient of 0.811, indicating good reliability. For descriptive purposes, overall QoL scores were categorized according to the distribution of the study sample as follows: poor QoL, score < (mean - 1 SD); average QoL, score between (mean - 1 SD) and (mean + 1 SD); and good QoL, score > (mean + 1 SD).

Data collection procedure

Before data collection commenced, local community representatives were informed about the objectives of the study. Eligible participants were approached at their residences by the principal investigator. The purpose of the study was explained in the local language, and written informed consent was obtained before enrolment. For participants who were unable to read because of illiteracy or visual impairment, the consent form was read aloud in Telugu before consent was obtained.

Face-to-face interviews were conducted using the structured questionnaire in a private setting whenever feasible to maintain confidentiality and minimize response bias. Participants who declined participation during the first visit were approached once more to confirm their decision. Individuals who continued to decline participation were considered nonresponders and were not contacted further.

Data quality assurance

The questionnaire was pretested before the initiation of the main survey to assess its clarity, comprehensibility, and feasibility of administration. Necessary modifications were incorporated before data collection. Completed questionnaires were reviewed daily for completeness and internal consistency before data entry. Data were coded and entered into Microsoft Excel using predefined coding formats. Interim data verification and consistency checks were performed regularly by the principal investigator to identify missing values, coding errors, and inconsistencies. Any discrepancies identified during the interim data review were verified against the original questionnaires and corrected before the final analysis.

Variables

The primary outcome variable was the overall QoL score measured using the WHOQOL-BREF questionnaire. Independent variables included age, sex, educational status, marital status, family type, occupation, source of income, socioeconomic status, enrolment in government welfare schemes for older adults, and participation in household decision-making.

Statistical analysis

Data were coded and entered into Microsoft Excel before being analysed using R version 4.4.1 through RStudio. Data management, transformation, and visualization were performed using the tidyverse meta-package, while summary tables were generated using the gtsummary package. Continuous variables were summarized using the mean and SD, whereas categorical variables were expressed as frequencies and percentages.

The normality of continuous variables was assessed using the Shapiro-Wilk test. Differences in mean QoL scores between two independent groups were evaluated using the independent-samples Student’s t-test, whereas comparisons involving three or more groups were performed using one-way ANOVA. Variables demonstrating statistically significant associations in univariable analyses and those considered biologically relevant were entered into a multiple linear regression model to identify independent determinants of overall QoL. Multicollinearity was assessed using variance inflation factors (VIFs), and model assumptions were evaluated using residual diagnostic plots. All statistical tests were two-tailed, and a p-value <0.05 was considered statistically significant.

Ethical considerations

The study protocol was approved by the Institutional Ethics Committee (IEC) of the participating medical college (Approval No. IEC-177/2023). Written informed consent was obtained from all participants before enrolment. Participation was voluntary, and participants were informed of their right to withdraw from the study at any stage without penalty. The confidentiality and privacy of participant information were maintained throughout the study by de-identifying all collected data and restricting access to study records to the research team.

## Results

A total of 400 community-dwelling older adults participated in the study. The majority of participants were aged 60-69 years (311, 77.8%), while only 21 (5.3%) were aged 80 years or older. Female participants constituted 234 (58.5%) of the study population. More than half of the participants were married (233, 58.3%), whereas 147 (36.8%) were widowed.

Educational attainment was generally low, with 233 (58.3%) participants being illiterate and only six (1.5%) having completed intermediate education or higher. Most participants belonged to joint families (227, 56.8%), while 166 (41.5%) lived in nuclear families and seven (1.8%) lived alone. Regarding economic characteristics, pensions (137, 34.3%) and family support (133, 33.3%) were the predominant sources of income, followed by current employment (90, 22.5%). The largest proportion of participants (162, 40.5%) belonged to the upper-lower socioeconomic class, while 146 (36.5%) belonged to the lower-middle class. Less than half of the participants (192, 48.0%) reported involvement in household decision-making, and only 128 (32.0%) were enrolled in at least one government welfare scheme for older adults (Table 2).

**Table 2:** Baseline characteristics of the study participants (N = 400). †Socioeconomic status was classified using the Modified Kuppuswamy Socioeconomic Scale (2023 update).

Characteristic	n (%)
Age group (years)	
60-69	311 (77.8)
70-79	68 (17.0)
80-89	14 (3.5)
≥90	7 (1.8)
Sex	
Male	166 (41.5)
Female	234 (58.5)
Marital status	
Single	14 (3.5)
Married	233 (58.3)
Married but separated	6 (1.5)
Widowed	147 (36.8)
Educational status	
Illiterate	233 (58.3)
Primary school	128 (32.0)
Middle or high school	33 (8.3)
Intermediate education or higher	6 (1.5)
Family type	
Nuclear	166 (41.5)
Joint	227 (56.8)
Living alone	7 (1.8)
Primary source of income	
Currently working	90 (22.5)
Pension	137 (34.3)
Family support	133 (33.3)
Other sources	40 (10.0)
Socioeconomic status†	
Upper	0 (0.0)
Upper middle	61 (15.3)
Lower middle	146 (36.5)
Upper lower	162 (40.5)
Lower	31 (7.8)
Participation in household decision-making	
Yes	192 (48.0)
No	208 (52.0)
Enrolment in a government welfare scheme for older adults	
Yes	128 (32.0)
No	272 (68.0)

The mean overall QoL score among the 400 participants was 37.03 ± 10.35, with scores ranging from 18.90 to 59.85. Among the four WHOQOL-BREF domains, the psychological domain had the highest mean transformed score (41.34 ± 13.14), followed by the environment (40.24 ± 11.42) and physical health (39.44 ± 13.12) domains. The social relationships domain had the lowest mean score (27.08 ± 8.88) (Table 3).

**Table 3:** WHOQOL-BREF domain scores among the study participants (N = 400). WHOQOL-BREF: World Health Organization Quality of Life-BREF questionnaire.

WHOQOL-BREF measure	Mean ± SD	Range
Overall quality of life	37.03 ± 10.35	18.90-59.85
Physical health	39.44 ± 13.12	18.28-66.28
Psychological health	41.34 ± 13.14	21.32-69.32
Social relationships	27.08 ± 8.88	16.00-58.64
Environment	40.24 ± 11.42	20.00-64.00

The distribution of overall QoL among the study participants is presented in Table 4. Most participants, 236 (59.0%), had average QoL, while 102 (25.5%) were categorized as having good QoL. Conversely, 62 (15.5%) participants were classified as having poor QoL.

**Table 4:** Distribution of overall quality of life among the study participants (N = 400).

Overall quality of life	n (%)
Poor	62 (15.5)
Average	236 (59.0)
Good	102 (25.5)

In bivariate analyses, the mean overall QoL score differed significantly by sex, participation in household decision-making, and enrolment in welfare schemes for older adults, based on the independent-samples Student’s t-test, and by educational status, primary source of income, and socioeconomic status, based on one-way ANOVA (Table 5). Female participants (234, 58.5%) had significantly higher mean QoL scores than male participants (166, 41.5%) (38.7 ± 10.6 vs. 35.8 ± 10.0; p = 0.006). QoL also differed significantly according to educational attainment, with participants who had completed middle or high school (33, 8.3%) reporting the highest mean score (51.4 ± 4.1; p < 0.001). Participants receiving income from pensions (137, 34.3%) or other sources (40, 10.0%) had higher QoL scores than those who were currently working (90, 22.5%) or dependent on family support (133, 33.3%) (p < 0.001). Similarly, participants belonging to the upper-middle (61, 15.3%) and lower-middle (146, 36.5%) socioeconomic classes had significantly higher QoL scores than those in the upper-lower (162, 40.5%) and lower (31, 7.8%) socioeconomic classes (p < 0.001). Participants involved in household decision-making (192, 48.0%) and those enrolled in government welfare schemes (128, 32.0%) also reported significantly higher QoL scores than their counterparts (both p < 0.001). No statistically significant differences in overall QoL were observed across age groups (p = 0.241), marital status (p = 0.611), or family type (p = 0.794).

**Table 5:** Association between participant characteristics and overall quality of life (N = 400). †One-way ANOVA. ‡Independent-samples Student’s t-test. Statistical significance was set at p < 0.05.

Characteristic	Overall QoL score, mean ± SD	p value
Age group (years)		
60-69	37.3 ± 10.4	0.241†
70-79	36.3 ± 10.5	
80-89	38.3 ± 11.1	
≥90	29.8 ± 3.0	
Sex		
Male	35.8 ± 10.0	0.006‡
Female	38.7 ± 10.6	
Marital status		
Single	36.0 ± 15.7	0.611†
Married	37.3 ± 10.6	
Married but separated	35.9 ± 1.0	
Widowed	36.7 ± 10.5	
Educational status		
Illiterate	31.8 ± 7.5	< 0.001†
Primary school	42.6 ± 9.5	
Middle or high school	51.4 ± 4.1	
Intermediate education or higher	35.5 ± 4.1	
Family type		
Nuclear	36.5 ± 10.1	0.794†
Joint	37.9 ± 10.3	
Living alone	21.0 ± 1.2	
Primary source of income		
Currently working	33.7 ± 9.1	< 0.001†
Pension	39.4 ± 9.1	
Family support	34.4 ± 10.2	
Other sources	45.1 ± 11.3	
Socioeconomic status		
Upper-middle	47.5 ± 10.8	< 0.001†
Lower-middle	40.5 ± 9.2	
Upper-lower	31.1 ± 6.5	
Lower	30.5 ± 5.9	
Participation in household decision-making		
Yes	41.6 ± 10.7	< 0.001‡
No	35.9 ± 9.8	
Enrolment in an elderly welfare scheme		
Yes	41.0 ± 9.1	< 0.001‡
No	35.2 ± 10.4	

Multiple linear regression analysis was performed to identify independent determinants of overall QoL after adjusting for potential confounding variables (Table 6). Increasing age emerged as an independent determinant of lower overall QoL (β = -0.13, p = 0.013). Compared with illiterate participants, those who had completed primary school (β = 3.67, p < 0.001) and middle or high school education (β = 11.93, p < 0.001) had significantly higher QoL scores, whereas intermediate education or higher was not a significant determinant of QoL (p = 0.065). Compared with participants in the lower socioeconomic class, those belonging to the upper-lower (β = 4.13, p = 0.007), lower-middle (β = 11.01, p < 0.001), and upper-middle (β = 15.21, p < 0.001) socioeconomic classes demonstrated significantly higher QoL scores. In addition, enrolment in government welfare schemes for older adults (β = 6.00, p < 0.001) and participation in household decision-making (β = 3.64, p < 0.001) emerged as independent determinants of higher overall QoL.

**Table 6:** Multiple linear regression analysis of factors associated with overall quality of life among the study participants (N = 400). Reference categories: illiterate, lower socioeconomic class, not enrolled in an elderly welfare scheme, and no participation in household decision-making. β, regression coefficient. Variables with p < 0.05 were considered statistically significant.

Variable	β coefficient	Standard error	p-value
Intercept	31.47	3.62	< 0.001
Age (years)	-0.13	0.05	0.013
Educational status			
Primary school	3.67	1.05	< 0.001
Middle or high school	11.93	1.53	< 0.001
Intermediate education or higher	-7.35	3.97	0.065
Socioeconomic status			
Upper-lower	4.13	1.53	0.007
Lower-middle	11.01	1.73	< 0.001
Upper-middle	15.21	1.74	< 0.001
Enrolment in an elderly welfare scheme (yes)	6	0.72	< 0.001
Participation in household decision-making (yes)	3.64	1	< 0.001

## Discussion

The present study provides an evaluation of QoL among 400 community-dwelling older adults in Visakhapatnam. As life expectancy increases globally, ensuring a high QoL among older adults is essential for promoting well-being and healthy ageing. The mean overall QoL score observed in this study was 37.03 (SD = 10.35), with 236 (59.0%) participants classified as having average QoL. This finding is broadly consistent with Punabaka NK et al. (2023), who reported predominantly moderate QoL among older adults residing in urban areas of Tirupati [8]. However, the QoL scores observed in the present study were lower than those reported by Ahmed MT et al. (2017) in Bengaluru (50.02) [9] and Sarkar A et al. (2023) in Bhubaneswar (47.52) [10].

In an international context, the findings of this study were also lower than those reported by Khan N et al. (2014) in Bangladesh (58.08) [11] and Wijesiri HS et al. (2023) in Sri Lanka (56.73) [12]. These regional and international differences may reflect variations in socioeconomic conditions, cultural attitudes towards ageing, and the availability of social support systems. Differences in study settings, participant characteristics, and sampling methods may also have contributed to these variations.

Domain-specific variations

Among the WHOQOL-BREF domains, the social relationships domain recorded the lowest mean score (27.08), compared with the physical health (39.44), psychological health (41.34), and environment (40.24) domains. This finding may reflect changes in family structures in India, where the transition from traditional joint families to nuclear families may have reduced opportunities for social interaction and intergenerational support among older adults. Similar observations have been reported by Jamuna D (2000), who highlighted the influence of changing family dynamics on the social well-being of older adults [13].

Sociodemographic determinants

Age

Although no statistically significant differences in QoL were observed across age groups in the univariable analysis, multiple linear regression identified increasing age as an independent determinant of lower QoL. Participants aged 90 years or older (7, 1.8%) reported the lowest mean score (29.8). The discrepancy between the univariable and multivariable analyses may be explained by the regression model treating age as a continuous variable, thereby preserving more statistical information than categorical age-group comparisons. Shah VR et al. (2017) provided related QoL data for an older urban population in Ahmedabad [7], while Devraj S and D'mello M (2019) identified age as a determinant of QoL among older adults in Mangalore [14], suggesting that advancing age may adversely affect physical functioning and overall well-being.

Sex

Female participants (234, 58.5%) reported significantly higher mean QoL scores than male participants (166, 41.5%) (38.7 vs. 35.8). Although several studies have reported lower QoL among women because of financial dependence and social disadvantages, Rao R et al. (2024) also identified female sex as an independent correlate of lower QoL [15]. The contrasting findings in the present study may reflect stronger family support and different coping mechanisms among women in this setting.

Education 

Educational attainment emerged as an important determinant of QoL. Mean QoL scores increased from 31.8 among illiterate participants (233, 58.3%) to 51.4 among those who had completed middle or high school (33, 8.3%). Although participants with intermediate education or higher (6, 1.5%) had a lower mean QoL score (35.5), this association was not statistically significant in the adjusted analysis. Similar observations have been reported by Rao R et al. (2024) [15].

Socioeconomic determinants and autonomy

Socioeconomic Status 

Socioeconomic status was an important determinant of QoL. Participants belonging to the upper-middle socioeconomic class (61, 15.3%) had the highest mean QoL score (47.5), whereas those in the lower socioeconomic class (31, 7.8%) had the lowest score (30.5). These findings are consistent with Gupta A et al. (2014), who reported that better socioeconomic status contributes to improved health, independence, and QoL among older adults [16].

Financial Independence 

Participants receiving pensions (137, 34.3%) or income from other sources (40, 10.0%) reported higher QoL than those who were currently working (90, 22.5%) or dependent on family support (133, 33.3%). Financial security through pensions and other stable income sources may improve autonomy and access to healthcare, consistent with the findings of Kumar SG et al. (2014) [17]. However, because of the cross-sectional nature of the study, the direction of this relationship cannot be established.

Autonomy 

Participants involved in household decision-making (192, 48.0%) reported significantly higher QoL than those who were not involved (208, 52.0%). Participation in family decision-making may enhance self-esteem, social inclusion, and psychological well-being, thereby contributing to better QoL among older adults. Nevertheless, it is equally plausible that older adults with better health and higher QoL are more likely to remain actively involved in household decision-making.

Strengths and limitations

The use of probability-based sampling and a validated WHOQOL-BREF instrument strengthens the methodological rigour and validity of the study. Owing to its cross-sectional design, causal relationships between the identified determinants and QoL cannot be established. As data were collected through face-to-face interviews, recall and social desirability biases may have influenced participants’ responses. Although interviewer bias was minimised through the use of a standardised interviewer-administered questionnaire and uniform data collection procedures, it cannot be completely excluded. The replacement of unavailable participants after repeated visits may have introduced selection bias. Some subgroups had relatively small sample sizes, which may have limited the precision of subgroup comparisons. Furthermore, the exclusion of severely ill older adults may have resulted in an underestimation of poor QoL, and the findings may not be generalisable beyond similar urban settings.

## Conclusions

This community-based cross-sectional study demonstrated that the majority of older adults residing in an urban field practice area had average QoL, while approximately one in six experienced poor QoL. Among the WHOQOL-BREF domains, the social relationships domain had the lowest mean score, indicating that social well-being remains an important challenge in this population. Educational attainment, socioeconomic status, enrolment in elderly welfare schemes, and participation in household decision-making emerged as important determinants of QoL, whereas increasing age was independently associated with lower QoL.

These findings highlight the importance of social and socioeconomic factors in relation to QoL among older adults. Strategies to promote healthy ageing should extend beyond clinical care to strengthen social support, improve access to welfare programmes, enhance educational and economic opportunities, and encourage the active participation of older adults in household and community decision-making. Future longitudinal, multicentre, and intervention studies are warranted to clarify temporal relationships, evaluate the effectiveness of such interventions, and further clarify the determinants of QoL in the ageing population.
